# Ternary optimization for designing metasurfaces

**DOI:** 10.1038/s41598-021-96564-5

**Published:** 2021-08-24

**Authors:** Azin Hojjati, Mohammad Soleimani, Vahid Nayyeri, Omar M. Ramahi

**Affiliations:** 1 School of Electrical Engineering, Iran University of Science and Technology, Tehran, 1684613114 Iran; 2 School of Advanced Technologies, Iran University of Science and Technology, Tehran, 1684613114 Iran; 3 Department of Electrical and Computer Engineering, University of Waterloo, Waterloo, N2L3G1 Canada

**Keywords:** Electrical and electronic engineering, Applied physics

## Abstract

A fully automated approach for designing metasurfaces whose unit cell may include metallic vias is proposed. Towards this aim, a ternary version of the particle swarm optimization (PSO) algorithm is employed in order to find the optimal metallic pattern and via-hole positions simultaneously. In the proposed design method, the upper surface of the unit cell is first pixelated. One of the possible three states of a metallic covered pixel, an uncovered etched pixel and a pixel containing a centered metalized via-hole is assigned to each pixel. The optimal state of each pixel is then determined by utilizing a ternary PSO algorithm to achieve favorable design goals. This method can be used for designing various metasurfaces as well as other via-assisted electromagnetic structures. As a proof of concept, the proposed method was applied to design two surfaces: a frequency selective surface with a minimum resonance frequency, and a linear-to-circular polarization converter with a maximum polarization conversion bandwidth. Comparison of the results with previous works confirms the efficiency and capability of the proposed method to design diverse metasurfaces in an automated fashion without the need for any theoretical or physical model.

## Introduction

In recent years, metasurfaces have been considered as a simple, inexpensive and efficient solution widely used in diverse electromagnetic applications such as frequency selective surfaces (FSS)^1^, polarization converters^2^, absorbers^3,4^, artificial magnetic conductors^5^, electromagnetic harvesters^6,7^, holograms^8^ and antennas^9,10^. These electrically thin structures commonly consist of a periodic array of unit cells which are composed by one or several metallic patterns on a single or multiple dielectric substrates. Therefore, the specifications and characteristics of the metasurfaces entirely depends on their unit cells.

The unit cells are conventionally designed using the classical methods such as the equivalent circuit model. However, optimizing the design parameters is inevitable to achieve the desired results. Hence, designers first produce a unit cell topology according to the classical methods and then utilize optimization techniques typically embedded in most CAD packages to achieve the required goals. The question that may arise is whether such design is the best that can possibly be achieved. Since the optimization is about changing the dimension of the designed model, not its shape and topology, the performance of the final optimized design highly depends on the initial design topology and so it may not be the best solution. To address this challenge, the automated unit cell topology design by means of the pattern optimization was introduced^11–18^. In this method, the shape of the unit cell (the pattern to be etched into each copper layer of a PCB) is optimized to achieve the best possible performance. This optimization is not about changing the dimensions of a designed model but rather about its shape. The required optimization procedure is essentially making a decision as to which parts of the pattering area are covered with metal and which parts are not (i.e., etched). This was achieved by first dividing the unit cell’s area into pixels and then applying a binary global optimization algorithm. The binary optimizer would assign one of two states to each pixel: one state refers to metalization, and the other to etching the metal. Since there is no need for a proper initial (modeled based) design, the procedure can be performed in a fully automated fashion.

The application of such a pattern design by applying pixelization and a binary optimization technique has yielded superior performance in various technologies such as high impedance surfaces^11,12^, frequency-selective surfaces^13^, radar cross-section reducers^14,15^, electromagnetic energy harvesting surfaces^16^, polarization converters^17^, absorbers^18^, sensors^19^, and decoupling elements between microstrip antennas^20^. So far, however, only binary versions of optimizations have been utilized in the automated unit cell design, which limits the applicability of this approach to a two-state design paradigm (i.e., the presence or absence of a metallic layer on each pixel). However, many electromagnetic structures have benefited from metallic via-holes in their topologies. For instance, metallic vias have been used to miniaturize the size of a FSS unit cell by increasing the inductance and capacitance of adjacent unit cells in a single sided structure^21^, and also by extending the length of a loop for a double sided surface^22^. A variety of dual band FSSs with different applications and operation frequencies can be realized by using a diverse number of via-holes companion with metallic patterns^23^. Metallized via-holes have been also proven to be able to modify the bandwidth of a linear to circular polarization converter^24^ as well as a linear to linear polarization rotator^25^ and also to extend the bandwidth and enhance the transmission coefficient of metasurfaces^26^. Since these types of structures require an optimization process to arrange via-holes, metallic, and empty pixels simultaneously, the binary optimization approaches would not be applicable.

In this paper, an automated design method is presented by means of a global ternary optimization algorithm. In this method, the surface of the unit cell is first pixelated. Then the ternary optimizer would assign one of the possible three states to every pixel: metalization, no metalization, and metallic via. After describing the design process using the proposed approach, its application is presented in two different examples.

## Design method development

A periodic array of a unit cell located on a dielectric substrate is considered as a metasurface with an arbitrary function. The upper face of the unit cell is composed of a metallic pattern which may contain a number of metalized via-holes. In order to design the unit cell topology automatically, the upper surface of the cell is first pixelated. One trit (ternary digit^27^) is assigned to each pixel which can opt one of the three possible states: 0, 1 or 2, representing an uncovered (etched) pixel, a metal covered pixel, and a pixel with a centered metalized via-hole, respectively. Thus, a string of trits with a length of the number of the pixels is formed. Subsequently, a ternary optimization is utilized to find the best possible state (0, 1, or 2) of each trit.

It should be noted that a larger number of pixels (*N*) results in a smaller pixel size, which leads to a finer detailed pattern that has stronger potential to achieve the design objective. On the other hand, by increasing the number of pixels, the optimization solution space (the number of possible structures) which equals to $3^N$ will increase dramatically and the optimization convergence rate may be reduced considerably. Therefore, a trade-off has to be considered between the number of the pixels and the convergence rate. In order to decrease the number of independent pixels, and thus to increase the convergence rate, different types of symmetry can be applied to the unit cell topology. Therefore, only a particular part of the unit cell would be pixelated and the entire pattern of the unit cell will be created by means of mirroring (or rotating) the pixelated area according to the symmetry type.

**Figure 1:**
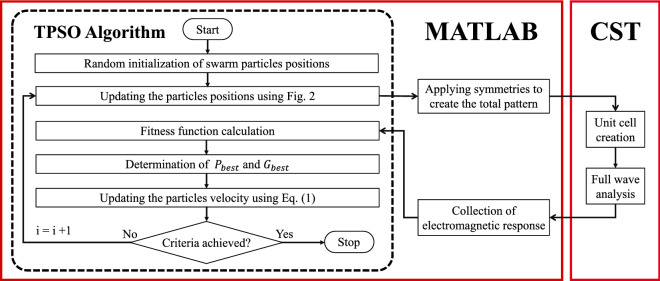
Flowchart of the TPSO-based unit-cell design procedure.

As shown in Fig. 1, each trit string, which represents a candidate solution of the problem, is transformed from the MATLAB environment to a unit cell in CST Microwave Studio. Then, by applying periodic boundary conditions, this unit cell is analyzed using the full-wave frequency-domain solver of CST, and the results are returned to MATLAB in order to calculate the cost function based on the desired goals. The calculated cost function is then delivered to the optimization algorithm to update the trit strings for next iteration. Once the optimization process stops by reaching the termination criteria, the optimized unit cell topology is obtained.

Although many different continuous and binary versions of optimization algorithms have been developed, ternary optimization algorithms have been introduced in a more limited way^28–30^. In this paper, a ternary version of the particle swarm optimization (PSO) namely TPSO, which was introduced in^30^ for an optimal switch placement in power distribution systems, is employed because of its ease of implementation. For an optimization problem with *N* optimization parameters, as well as the traditional PSO algorithm, a number of candidate solutions (particles) composing the population (swarm), explore the *N*-dimensional search space by moving around it. Therefore, two *N* dimensional vectors of $X_m$ and $V_m$ are assigned to each swarm particle position and velocity, respectively. Unlike the traditional PSO algorithm in which the particle position vector elements can possess continuous values, in this algorithm, only one of the three basic phasor values of $1\angle -120^\circ$, $1\angle 0^\circ$ or $1\angle 120^\circ$ can be assigned to these elements. Each particle position vector element is initialized randomly by one of these basic phasor values, and the initial value of the velocity vectors’ elements is zero.

During the algorithm iterations, each particle’s velocity (the *m*th particle as an example) is adjusted according to the swarm’s and its own best experiences as,

1 $$\begin{aligned} V_m^t = V_m^{t - 1} + {c_1}{e_1} \cdot P_m^{t - 1} + {c_2}{e_2} \cdot {G^{t - 1}}, \end{aligned}$$

where the superscript $^t$ indicates the $\mathrm {t}$th iteration of the algorithm, $P_m = [p_{m,1},p_{m,2},\ldots ,p_{m,N}]$ and $G = [g_1, g_2,\ldots , g_N]$ are the best experiences of the *m*th particle and the swarm, respectively, $c_1$ and $c_2$ are positive constants, and $e_1$ and $e_2$ are vectors with random elements between 0 and 1 to guarantee the random behavior of the optimization algorithm. It should be noted that (1) is a modified version of the velocity update equation in the traditional PSO algorithm^31^.

While in (1), $V_{m,n}$ assumes continuous complex values, the position vectors are ternary-valued (i.e.,$X_{m,n}$ is $1\angle -120^\circ$, $1\angle 0^\circ$ or $1\angle 120^\circ$). Therefore, to update the position vectors, a mapping between the continued-valued velocities to the ternary-valued positions is required. To this end, for each particle, each velocity vector element ($V_{m,n}$) with a phase in the range of $[-180, 180]$ is linearly mapped to a [0, 1] interval according to

2 $$\begin{aligned} V_{m,n}^\prime = \frac{\angle {V_{m,n}} + 180}{360}, \end{aligned}$$

which results in three basic mapped values of 1/6, 3/6 and 5/6 for the three basic phasor states of $1\angle -120^\circ$, $1\angle 0^\circ$ and $1\angle 120^\circ$, respectively.

**Figure 2:**
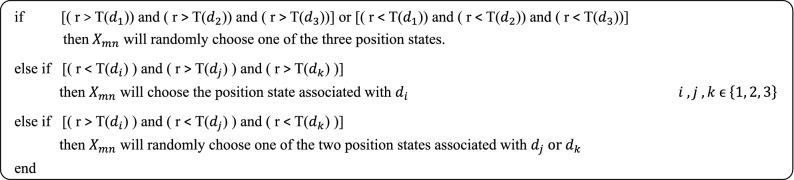
The procedure of updating particles' position in the TPSO algorithm.

Each mapped value ($V^\prime$) is then subtracted from the three basic mapped values of 1/6, 3/6 and 5/6 to respectively produce distances of $d_1, d_2,$ and $d_3$. In order to avoid the algorithm being trapped in local minimums, these distances are then transformed to another [0, 1] interval by using the following transformation function

3 $$\begin{aligned} {T}({d_k}) = {1 - \exp \left( {1 - \frac{1}{{c ~ {d_k}}}} \right) }, \end{aligned}$$

where $k= 1,2,3$, and *c* is a constant coefficient chosen equal to 3 so that the optimization algorithm represents a good performance^30^. Then, by choosing a random number (r) between 0 and 1, and comparing it to $T(d_k)$, the particle positions are updated according to the algorithm, shown in Fig. 2.

It should be noted that the aforementioned process is applied to every $V_m$ so that all $X_m$ vectors are updated.

To our best knowledge, no study has been performed to investigate the performance of the aforesaid TPSO algorithm so far. Therefore, to ensure the applicability of this algorithm in finding the global optimum and its appropriate convergence, and also in order to find a proper range for the swarm population as well as the maximum iteration number, this algorithm was examined using several standard benchmark functions (see the “Appendix”).

## Design examples

In order to demonstrate the applicability of the proposed method, it was applied to design two metasurfaces with different functions. The design details and results are provided in the following subsections.

### Miniaturized bandstop FSS

As the first example, the proposed optimization method is used to design a transmissive bandstop FSS with the lowest possible resonant frequency for a unit cell with specified constant size. The unit cell was based on an ungrounded (non-metal backed) FR-4 substrate with $\varepsilon _r=4.3$ and a standard thickness of 1.6 mm and a side length of $P=9.1$ mm.

**Figure 3:**
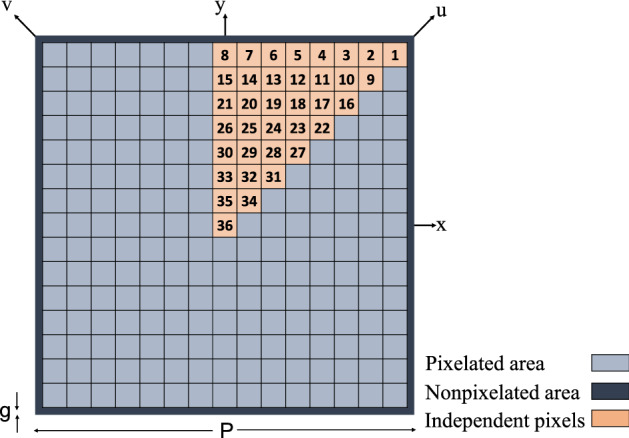
Schematic of the pixelated FSS unit cell.

**Figure 4:**
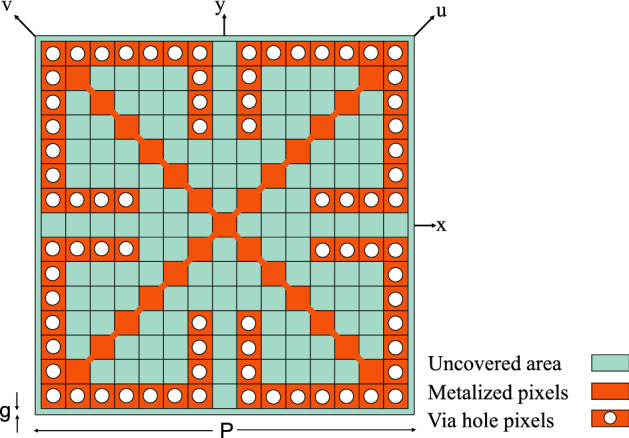
Schematic of the optimized FSS unit cell.

The upper surface of the substrate for the unit cell is pixelated. Each square pixel has a side length of 0.6 mm in order to be able to enclose a metalized via-hole with a hole diameter of 0.3 mm and an annular ring size of 0.15 mm (these dimensions are constrained by fabrication limitations). On the other hand, the maximum pixel size has to be chosen so that fine patterns with acceptable details could be fabricated. In order to decrease the number of independent pixels which leads to an increase in the convergence rate and also to reduce the FSS sensitivity to the polarization of the incident wave, an eight-reflection symmetry was applied to the unit cell. Accordingly, only 36 numbered pixels are included in the optimization process (see Fig. 3). The full pattern of the unit cell would be formed by reflecting the one-eighth pattern across the horizontal, vertical and diagonal axes. A non-pixelated area with a width of $g= 0.05$ mm was maintained around the unit cell to avoid metal contact between adjacent unit cells.

A population was considered with a swarm of 50 particles in the TPSO algorithm where the position vector of each particle is a 36-trit string. Each trit is first initialized by one of the three 0, 1 or 2 states (corresponding to basic phasor states of $1\angle -120^\circ$, $1\angle 0^\circ$ and $1\angle 120^\circ$) randomly. In order to achieve the lowest possible resonant frequency, the cost function (*CF*) is defined as:

4 $$\begin{aligned} CF =\frac{1}{f_0}, \end{aligned}$$

where $f_0$ indicates the first resonant frequency of the FSS, which is determined from the amplitude of the transmission coefficient ($|S_{21}|$).

After approximately 500 iterations, the TPSO algorithm converged and the optimized unit cell was achieved as shown in Fig. 4. It should be noted that in Fig. 4, the connection between pixels, which are connected diagonally from just one point in their corners, is slightly widened to prevent two pixels from being fragmenting during the fabrication process.

**Figure 5:**
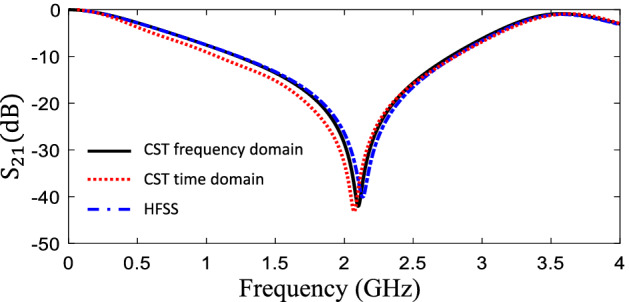
Transmission coefficient amplitude of the designed FSS.

The transmission coefficient of the proposed unit cell is shown in Fig. 5, which represents a band-stop behavior with a resonant frequency of about 2.1 GHz. In order to verify the simulation results obtained from CST frequency-domain solver, which was employed during the optimization procedure, the proposed structure was finally analyzed using the the CST time-domain and HFSS full-wave electromagnetic simulation tools. The results are compared in Fig. 5, demonstrating strong agreement between them.

In order to investigate the performance and objectives of our optimized design (miniaturization of the unit cell or reducing the resonant frequency for a unit cell with constant dimensions,) a comparison is made between the designed FSS using the proposed method and those in recent similar works. Two FSS unit cells were designed in^21^ and^22^, with the objectives of having sizes of $0.0715\times 0.0715~\lambda _0^2$ and $0.0625 \times 0.0625~\lambda _0^2$, respectively, where $\lambda _0$ is the free-space wavelength corresponding to their first resonant frequency. The dimensions of our FSS unit cell is $0.0637 \times 0.0637~\lambda _0^2$ which is considerably smaller than the designed unit cell in^21^ and almost equals to that in^22^. It is noteworthy that the unit cell introduced in^22^ was designed by means of two metallic layers (on both sides of a substrate), while the unit cell designed in this work and the one in^21^ only uses a single metallic layer on the upper face of the substrate. It is also worth mentioning that the designed unit cell in this work has a topology similar to the one in^21^. Although our proposed unit cell is smaller, the main difference is that in previous works, the unit cell was designed based on employing an equivalent circuit model, while in this paper, the unit cell was designed using a fully automated optimization procedure, which does not call for physical models and analytic methods, while being completely independent of any initial design. This design automation feature is a critical advantage of the method introduced in this paper in comparison to previous works.

### Wideband reflective linear to circular polarizer

The purpose of this example is to design a reflective linear to circular polarization converter aiming to maximize the polarization conversion bandwidth. A grounded (metal backed) RO4003C substrate with $\varepsilon _r=3.55$, loss tangent of 0.0027, and a standard thickness of 60 mil was used to provide a reflective surface. The side length of the square unit cell was set to $P=9.8$ mm according to a previous work^32^. The upper surface of this unit cell was pixelated and each pixel has a dimension of 0.7 mm in order to be able to enclose a metalized via-hole with a hole diameter of 0.3 mm and an annular ring size of 0.15 mm. As mentioned previously, the minimum pixel size is limited by the fabrication constraints and the maximum pixel size is defined by the minimum acceptable details of a pattern.

**Figure 6:**
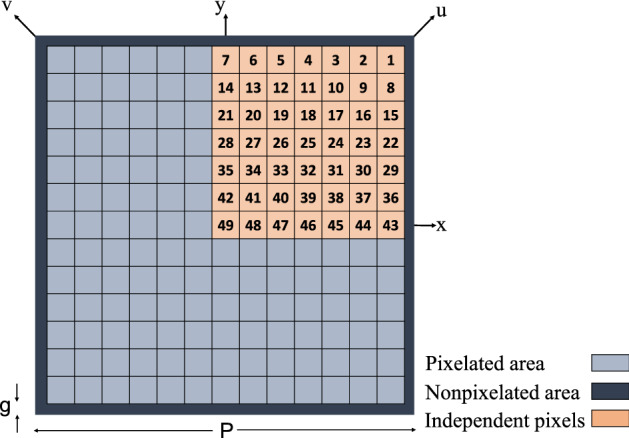
Schematic of the pixelated polarization converter unit cell.

By assuming an incident plane wave with a linear polarization along a diagonal axis (the *v* axis shown in Fig. 6), for a linear to circular polarization conversion, the unit cell has to behave differently for each of the two horizontal and vertical wave components of the incident field since there is a need for a 90$^{\circ }$ phase difference between the reflected x- and y-polarized waves. Thus, the diagonal symmetry cannot be applied to the unit cell and only vertical and horizontal symmetries (along *x* and *y* axes) are applicable. Therefore, a four-reflection symmetry is considered for the unit cell to reduce the number of independent pixels, which participate in the optimization process, to 49, as shown in Fig. 6. A border area with a width of $g=0.35$ mm was not pixelated to ensure the adjacent unit cells are not in contact with each other.

**Figure 7:**
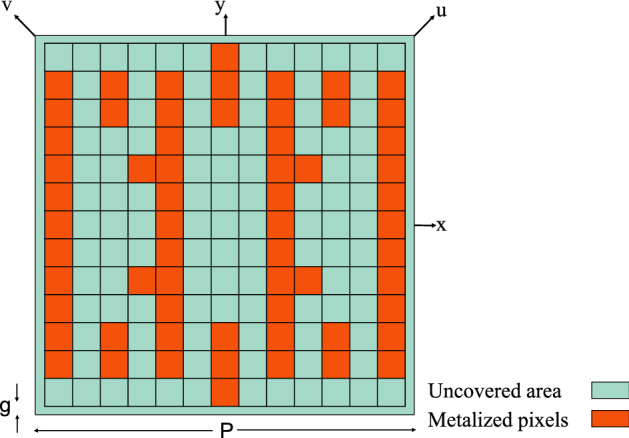
Schematic of the optimized polarization converter unit cell.

In the TPSO algorithm, a number of 70 swarm particles were considered, where a 49-trit string represents the position vector of each particle. Every trit was first initialized randomly by one of the three 0, 1 or 2 values. In order to achieve a maximum polarization conversion bandwidth, the cost function was defined as:

5 $$\begin{aligned} CF = \frac{1}{FBW}, \end{aligned}$$

where *FBW* represents the fractional bandwidth of the polarizer in which the axial ratio (AR) is below 3 dB (because a wave having an AR lower than 3 dB is commonly known as a circularly polarized wave).

During the iterations of the TPSO, to calculate the cost function, each candidate unit cell was simulated using the CST frequency-domain solver, where periodic boundary conditions were applied along the *x* and *y* directions. The reflection coefficient’s amplitude and phase were obtained along the *u* and *v* axes. The AR is then calculated by^33^:

6 $$\begin{aligned} AR= \sqrt{\dfrac{|\Gamma _{u}|^2 + |\Gamma _{v}|^2 +\sqrt{|\Gamma _{u}|^4 + |\Gamma _{v}|^4 + 2|\Gamma _{u}|^2 |\Gamma _{v}|^2\cos {2\Delta \varphi }}}{|\Gamma _{u}|^2 + |\Gamma _{v}|^2 -\sqrt{|\Gamma _{u}|^4 + |\Gamma _{v}|^4 + 2|\Gamma _{u}|^2 |\Gamma _{v}|^2\cos {2\Delta \varphi }}}}, \end{aligned}$$

where $|\Gamma _{u}|$ and $|\Gamma _{v}|$ are the amplitudes of the reflected coefficient’s components along the *u* and *v* axes, respectively, and $\Delta \varphi$ indicates the phase difference between these two components.

**Figure 8:**
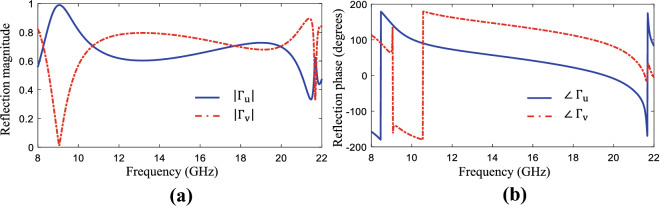
Reflection coefficient (**a**) amplitude and (**b**) phase of the optimized polarization converter surface.

**Figure 9:**
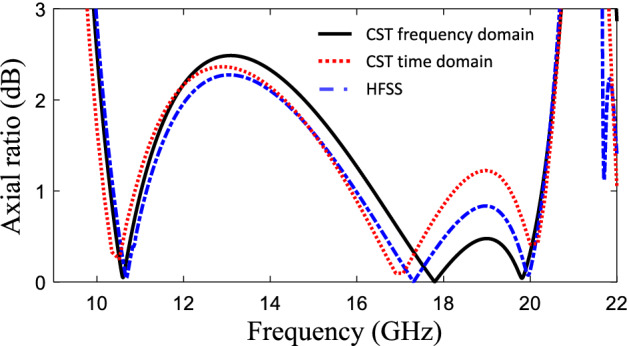
Axial ratio of the designed polarization converter surface.

**Table 1:** Standard benchmark functions and their minimum values.

Benchmark name	Benchmark function’s formula where $X = [x_1,x_2, \ldots ,x_{N}]$	Function’s minimum for $x_i\in \{0,1,2\}$ and $N=20$
Sphere function	$f(X) = \sum \limits _{i = 1}^N {x_i^2}$	0
Step function	$f(X) = \sum \limits _{i = 1}^N {{{\left( {{x_i} + 0.5} \right) }^2}}$	5
Noise function	$f(X) = \sum \limits _{i = 1}^N {i.x_i^4 + r~}$, where *r* is a random number between [0, 1)	[0, 1)
Schwefel 1.6 function	$f(X) = \sum \limits _{i = 1}^N { - {x_i}\sin \left( {\sqrt{\left| {{x_i}} \right| } } \right) }$	− 39.5106
Schwefel 2.22 function	$f(X) = \sum \limits _{i = 1}^N {\left| {{x_i}} \right| } + \prod \limits _{i = 1}^N {\left| {{x_i}} \right| }$	0
Rastrigin function	$f(X) =10N + \sum \limits _{i = 1}^N {x_i^2 - 10\cos (2\pi {x_i})}$	0
Griewank function	$f(X) = 1 + \sum \limits _{i = 1}^N {x_i^2 /4000} - \prod \limits _{i = 1}^N {\cos \left( x_i/{\sqrt{i} }\right) }$	0
Rosenbrock function	$f(X) = \sum \limits _{i = 1}^N {\left[ {{{\left( {{x_{i + 1}} - x_i^2} \right) }^2} + {{\left( {100 - {x_i}} \right) }^2}} \right] }$	0
Ackley function	$f(X) = -20\exp \left( { - 0.2\sqrt{\frac{1}{N}\sum \limits _{i = 1}^N {x_i^2} } } \right) - \exp \left( {\frac{1}{N}\sum \limits _{i = 1}^N {\cos (2\pi {x_i})} } \right)$ $+ 20 + \exp (1)$	0

The optimization algorithm converged after approximately 600 iterations resulting in the optimized unit cell topology shown in Fig. 7. It is noteworthy that despite the possibility of all three states of choosing uncovered, metal covered and via-hole pixels in the optimization process, no via-hole has appeared in the optimized topology from the TPSO algorithm. The reflection coefficient amplitude and phase along the *u* and *v* axes for the periodic structure composed by the proposed unit cell is shown in Fig. 8, and the AR of the reflected wave from the array is shown in Fig. 9. It is observed in Fig. 8 that the designed metasurface provides a polarization conversion (3-dB AR) fractional bandwidth of 73% in a frequency rage of 9.7–20.9 GHz. In addition, a perfect linear to circular polarization conversion occurs at three frequencies: 10.6 GHz, 17.8 GHz and 19.8 GHz (a 0-dB AR since the two orthogonal reflection coefficient components along the *u* and *v* axes have a phase difference exactly equal to 90$^{\circ }$ and equal amplitudes). In order to verify the simulation results, in addition to the CST frequency-domain solver which was the main analyzer used in the optimization process, the AR of the reflected wave from the proposed surface was obtained by using the CST time-domain and HFSS solvers as well. The results are compared in Fig. 9, demonstrating a strong agreement between the results obtained using the three full-wave electromagnetic simulation tools.

We observe that the topology of the optimized unit cell is highly similar to that of the traditional strip shaped reflective linear to circular polarizer introduced in earlier work^32^. This strong similarity indicates the strong capability of our design procedure, despite random initialization, which resulted in a unit cell topology highly similar to a unit cell obtained by a physical model. Additionally, the 3-dB AR fractional bandwidth resulting from the topology obtained by our design method is approximately 73% while the bandwidth reported in the previous work is approximately 60%.

Finally, it is worth mentioning that in the proposed optimization-based design method, almost the whole optimization time is dedicated to full-wave electromagnetic simulations of unit cells. In both presented examples, using a machine equipped with an AMD RYZEN THREADRIPPER 1950X processor, simulation of every unit cell (i.e., every candidate solution of the problems) took about 100 seconds using 500 MB of memory. Notice that in the proposed method, parallel simulation of several unit cells (up to the swarm population) is possible, which can decrease the total optimization time appreciably. Furthermore, the required memory for compiling the TPSO algorithm in MATLAB was about 100 MB.

## Conclusion

A fully automated method for designing metasurfaces based on a ternary optimization algorithm was introduced. In this method, the surface of the unit cell is pixelated. However, unlike binary optimizers, in the ternary optimzer, a TPSO algorithm is implemented where one of three states is assigned to each pixel: an uncovered pixel, a metal covered pixel, or a pixel containing a metallic via-hole. As a proof of concept, the proposed method was applied in two design examples: a bandstop FSS that achieves lowest resonant frequency, and a reflective linear to circular polarization converter with widest polarization conversion bandwidth. In the two examples, the comparison with previous works demonstrated that the proposed method is capable to automatically produce an optimum design for the unit cell, thereby satisfying the desired design goals without the need for any physical or theoretical model, or even an initial design topology.

## Figures and Tables

**Figure 10:**
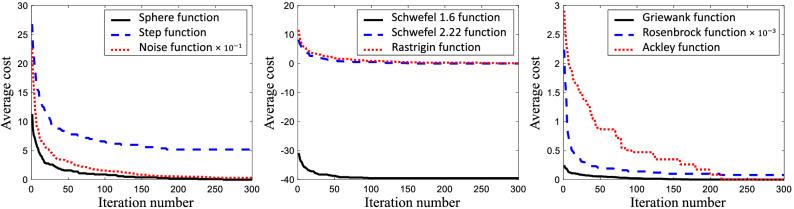
Benchmark function averaged values for 10 independent execution of a 40-particle swarm population with 20 parameters and 300 iterations.

## Appendix

### TPSO algorithm evaluation

The ability of the utilized TPSO algorithm in achieving the global minima of several standard benchmark functions shown in Table 1 was investigated. To this purpose, an optimization problem with optimization parameter number of $N=20$ was considered. A population of $M = 40$ particles was produced and a 20-element vector with randomly initialized elements by one of the three 0, 1 or 2 states was assigned to the position of each particle. The algorithm was then applied to each of these functions with 300 maximum number of iterations and the results were obtained by averaging the results of 10 independent executions. As it can be seen in Fig. 10, the TPSO algorithm is capable to reach the global minimum for each benchmark function properly, which its exact value is given in the last column of Table 1. It should also be noted that these benchmark functions include different types of local minimums in addition to the global ones. Therefore, achieving an acceptable convergence rate and the proximity of the obtained results from TPSO to the exact minimum values shown in Table 1 demonstrate a desirable global optimizing performance for this algorithm.

The TPSO algorithm functionality was also examined for different population numbers (M) and optimization dimensions (N). This investigation illustrates that for a population number of *N* to 2*N* (i.e., $N< M < 2N$)and a maximum iteration of more than 10*N*, there would be a high probability of the convergence to the global optimum.
